# Prognostic Significance of Sentinel Lymph Node Mapping in Merkel Cell Carcinoma: Systematic Review and Meta-Analysis of Prognostic Studies

**DOI:** 10.1155/2014/489536

**Published:** 2014-05-26

**Authors:** Ramin Sadeghi, Zohreh Adinehpoor, Masoud Maleki, Babak Fallahi, Luca Giovanella, Giorgio Treglia

**Affiliations:** ^1^Nuclear Medicine Research Center, Mashhad University of Medical Sciences, Mashhad 9176699199, Iran; ^2^Cutaneous Leishmaniasis Research Center, Mashhad University of Medical Sciences, Mashhad 9137913316, Iran; ^3^Research Institute for Nuclear Medicine, Tehran University of Medical Sciences, Tehran 1411713137, Iran; ^4^Department of Nuclear Medicine and PET/CT Center, Oncology Institute of Southern Switzerland, Via Ospedale 12, 6500 Bellinzona, Switzerland

## Abstract

*Aim.* To assess through a systematic review and meta-analysis of the literature the prognostic implication of sentinel lymph node mapping in Merkel cell carcinoma (MCC). *Materials and Methods.* PubMed and SCOPUS databases were searched by using “Merkel AND sentinel” as keywords. All studies with prognostic information regarding SLN mapping in cN0 MCC patients were included. Hazard ratio (HR) for overall survival (OS) and disease free survival (DFS) was used as effect size. *Results.* SLN biopsy predicted better DFS and OS as compared to the nodal observation in cN0 MCC patients (pooled HR for DFS: 1.61 (95% CI: 1.05–2.46), *P* = 0.028; pooled HR for OS: 1.08 (95% CI: 0.55–2.10), *P* = 0.8). Pathologically negative SLN (SLN−) patients had better OS (pooled HR: 4.42 (95% CI: 1.82–10.7), *P* = 0.0009) and DFS (pooled HR: 2.58 (95% CI: 1.78–3.73)) as compared to SLN+ patients. *Conclusion.* SLN mapping can provide strong prognostic information regarding OS and DFS in cN0 MCC patients. More importantly, SLN mapping can improve DFS and possibly OS in cN0 MCC patients as compared to nodal observation. As MCC is a rare tumor, large multicenter prospective studies are still needed to validate the survival benefit of SLN mapping.

## 1. Introduction

Merkel cell carcinoma (MCC) is a rare cutaneous neuroendocrine malignancy with high propensity for regional lymph node spread and recurrence [1]. Its origin is believed to be the primitive epidermal stem cells capable of epithelial or neuroendocrine differentiation [2]. This tumor is aggressive with high mortality and poor prognosis. However, the clinical course of MCC can be more aggressive in males, elderly patients, large tumors, and immunocompromised individuals [3, 4]. One of the most important prognostic factors in clinically node negative (cN0) MCC patients is the presence of occult regional lymph nodal involvement [2, 4–6].

Sentinel lymph node (SLN) mapping is an important and accurate technique for regional lymph node staging in many solid tumors [7–9]. By identifying the first lymph node in a nodal basin that receives the lymph flow from a solid tumor, SLN biopsy allows for careful pathologic evaluation of one or a few SLNs (instead of the whole regional basin) that are most likely to be pathologically involved.

There are several studies which reported the prognostic importance of positive SLN in MCC. It is reported that patients with positive SLN have 3 times higher relapse rate and 2 times higher disease specific mortality as compared to negative SLN patients [10, 11]. However, the literature is heterogeneous in this regard and several authors did not find statistical significant association between positive SLN pathology and recurrence or survival [4].

More importantly, a number of studies reported that patients with regional lymph node metastasis have better survival and lower recurrence when treated with lymphadenectomy or regional radiation therapy [12, 13]. It seems that early detection of regional nodal involvement in MCC patients by SLN biopsy can improve survival due to the early start of more aggressive treatment plans in the disease course [14]. However, other studies showed no survival benefit by SLN mapping in MCC and no consensus has been reached in this regard [15].

In the current study, we aimed to assess through a systematic review and meta-analysis of the literature the prognostic implication of sentinel lymph node (SLN) pathological status in patients with MCC evaluating whether SLN mapping may improve survival in cN0 MCC patients.

## 2. Material and Methods

### 2.1. Search Strategy

We followed the PRISMA guidelines for performing the current systematic review and meta-analysis (http://www.prisma-statement.org). We searched PubMed and SCOPUS databases using the following search algorithm: “merkel AND sentinel.” The literature search was performed by two authors independently and the last search was done on January 2014 without language or time limit. The reference lists of the relevant studies were reviewed for possible missing citations.

### 2.2. Inclusion Criteria

Studies which met one of the following criteria were included in the systematic review:

1. evaluation of prognostic value of pathologic involvement of SLN for determination of overall survival (OS) or disease free survival (DFS) in cN0 MCC patients;
2. evaluation of the prognostic value of SLN mapping in cN0 MCC patients for improvement of OS or DFS.

In addition, we collected individual patient data from the reported cases in the literature with enough prognostic information (at least pathological involvement of the SLN and time to events such as death or recurrence should be reported) in order to perform an individual patient meta-analysis. Letters to the editor and review articles were excluded. Studies without enough prognostic information were also excluded. Two authors reviewed independently the retrieved articles. All discrepancies were resolved by the third author opinion. Possible duplicate publications were discussed and only the most recent reports were considered.

### 2.3. Data Abstraction

Two authors independently performed the data abstraction, and data on authors, affiliations, publication date, SLN mapping method, patient characteristics, and quality of the included studies were retrieved. The main effect size we used in the current analysis was hazard ratio (HR) of DFS and/or OS which were either extracted directly from the included studies or estimated from survival curves as recommended by Parmar et al. [20]. We reported pooled values with 95% confidence intervals (95% CI). Oxford center for evidence-based medicine checklist for prognostic studies was used to assess the quality of the included studies [21].

### 2.4. Statistical Analyses

Dersimonian and Laird method (random effects model) was used to pool the HR among the studies [22]. The pooled results were expressed graphically by forest plots. Cochrane* Q* test was used for heterogeneity evaluation (*P* < 0.05 was considered statistically significant). The inconsistency (*I* ^2^) index was used to quantify the heterogeneity among the studies.

For publication bias evaluation, funnel plots and Egger's regression intercept were used [23]. All statistical analyses were performed by using Comprehensive Meta-analysis (version 2, Biostat Inc., USA) and SPSS (version 11.5, SPSS Inc., USA).

## 3. Results

**Figure 1:**
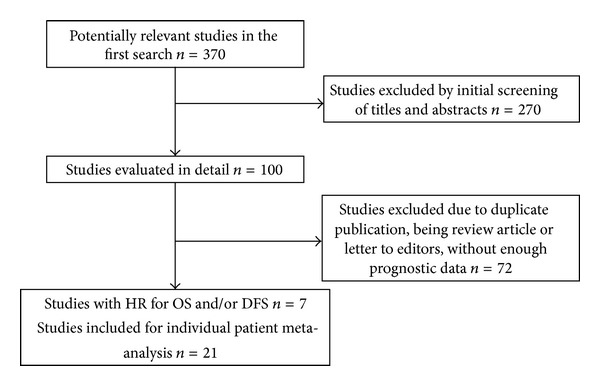
PRISMA flowchart of the study.

Figure 1 shows the PRISMA flowchart of the study. Four studies (1417 patients) provided HR of DFS and/or OS for SLN mapping as compared to other regional treatments in cN0 MCC patients [3, 14, 17, 18]. One of the included studies compared SLN mapping with all other treatments including clinical observation and lymph node dissection [3]. The other three compared the SLN mapping with observation in cN0 MCC patients.

Three studies (883 patients) provided HR of DFS and/or OS for pathologic condition of harvested SLN [15, 16, 19].

Three studies had prognostic information based on the Surveillance, Epidemiology, and End Results (SEER) database [4, 14, 16]. One of them had duplicate information and it was excluded [4]; therefore two articles were included in the systematic review [14, 16].

The characteristics of the included studies and quality assessment are shown in Table 1.

Twenty-one studies including 172 patients had prognostic information regarding pathological condition of SLN [10, 24–43]. Three studies had duplicate cases and were excluded [44–46]. We used cox regression model to analyze these cases and HR of OS and DFS for cN0 MCC patients with pathologically involved SLN as compared to pathologically noninvolved nodes were 6.13 (95% CI: 1.97–19.07) and 2.25 (95% CI: 1.16–4.33), respectively. Detailed survival analyses of these 172 patients are shown in Table 2.

### 3.1. Prognostic Importance of SLN Mapping versus Other Nodal Treatment Strategies

**Figure 2:**
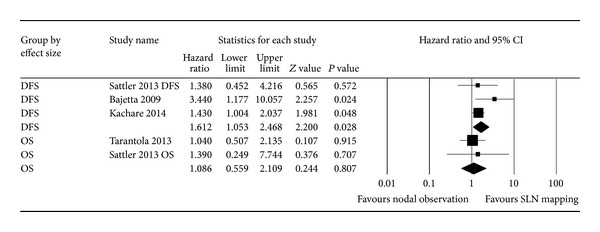
Forest plot of the hazard ratio (HR) of disease free survival (DFS) and overall survival (OS) for operative staging with SLN mapping versus nodal observation.

Quantitative synthesis is shown in Figure 2. Operative nodal staging with SLN biopsy predicted better DFS as compared to the nodal observation in cN0 MCC patients (pooled HR: 1.61 (95% CI: 1.05–2.46), *P* = 0.028, Cochrane* Q* value = 2.36, *P* = 0.3,* I* ^2^ = 15.3%). Nodal staging with SLN biopsy also predicted a slightly better OS, although the pooled HR (1.08 (95% CI: 0.55–2.10), Cochrane* Q* value = 0.093, *P* = 0.76,* I* ^2^ = 0%) was not statistically significant (*P* = 0.8).

### 3.2. Prognostic Importance of SLN Pathologic Status for Prediction of DFS and OS

**Figure 3:**
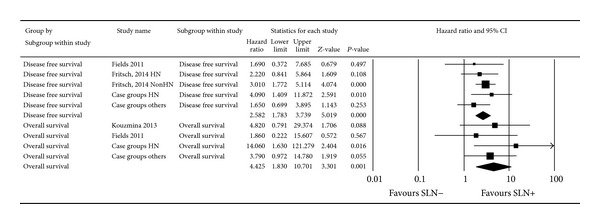
Forest plot of the hazard ratio (HR) of disease free survival (DFS) and overall survival (OS) for pathological SLN status.

Quantitative synthesis is shown in Figure 3. Pathologically negative SLN (SLN−) patients had better OS (pooled HR: 4.42 (95% CI: 1.82–10.7), *P* = 0.0009, Cochrane* Q* value = 1.8, *P* = 0.61,* I* ^2^ = 0%) and DFS (pooled HR: 2.58 (95% CI: 1.78–3.73), *P* = 0.000001, Cochrane* Q* value = 2.47, *P* = 0.64,* I* ^2^ = 0%) as compared to SLN+ patients.

**Figure 4:**
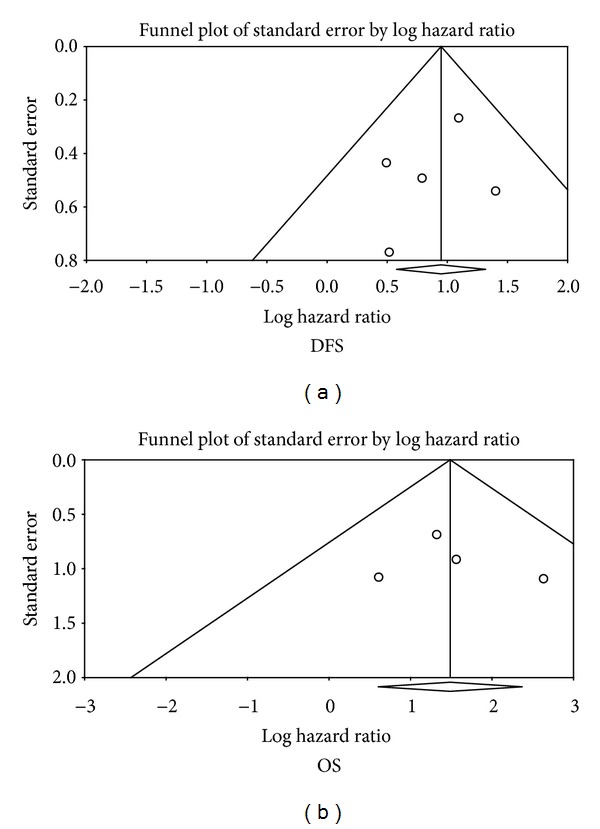
Funnel plots of meta-analyses of pathological SLN status for DFS and OS.

Funnel plots of OS and DFS meta-analyses are shown in Figure 4. Egger's regression intercepts for DFS and OS meta-analyses were −0.79 (*P* = 0.52) and 0.82 (*P* = 0.76), respectively.

Subgroup analysis regarding location of MCC did not show any difference between head and neck and other parts of the body regarding prognostic importance of SLN pathologic status (pooled HR of DFS for head and neck and other parts of the body were 2.92 (95% CI: 1.42–6), *P* = 0.003, and 2.54 (95% CI: 1.62–4), *P* = 0.000048, resp.).

## 4. Discussion

SLN mapping is an integral part of treatment in melanoma and breast cancer patients [47–49]. SLN mapping has been used to evaluate MCC for a long time and prognostic significance of this technique has been assessed in several reports. Recently, data of SEER database have been published regarding prognostic importance of SLN mapping in MCC, increasing our understanding in this regard.

In the current study, we reviewed the medical literature for two purposes: (1) to determine the prognostic importance of SLN status in MCC and (2) to assess the prognostic impact of SLN mapping versus other nodal treatments (mainly nodal observation) in MCC.

### 4.1. Prognostic Significance of SLN+ Status in MCC

MCC is a rare aggressive cutaneous tumor. Accurate staging of MCC patients is highly important for proper treatment planning. Radiological examinations such as PET imaging and CT have been used for this purpose with various results [50].

Positive lymph node disease at presentation is a strong indicator of poor outcome and reduces the 5-year survival rate to less than 50% [11].

Although medical literature is rich regarding SLN mapping in MCC [27, 33], studies with true survival analysis for the importance of SLN mapping are scarce. Overall, 3 studies had appropriate survival analyses and they were included in the current systematic review. We also gathered data of 172 cases from the medical literature and performed an individual patient meta-analysis.

Our meta-analysis showed that SLN status is a strong predictor of OS and DFS in MCC patients (pooled HR of 4.42 and 2.58, resp.). In other words, SLN+ patients may suffer MCC- related death and recurrence 4.42 and 2.58 times more frequently per unit time than SLN− patients. It is worth mentioning that, only in the study of Fritsch et al. [16], SLN status was a statistically significant predictor of survival. The other two studies by Kouzmina et al. [19] and Fields et al. [15] showed statistically nonsignificant results despite HR > 1 which denotes the lower MCC-related death or recurrence in the SLN− as compared to the SLN+ patients. The reasons of statistically nonsignificant results in these studies are most likely the low sample size and statistical power. Our meta-analysis by combining the results of individual studies increased the statistical power and yielded statistically significant results. Anyhow, the direction of effect size (HR) in the included studies is all the same and denotes the survival benefit of SLN− status. Location of the MCC is an important issue which has been brought up in the SLN mapping of MCC. Fritsch et al. [16] reviewed the information of SEER database and reported that SLN+ status was not an independent prognostic factor for predicting DFS. They attributed this finding to different lymphatic pathways and behavior in the head and neck area as compared to the other parts of the body [16, 51]. However, our meta-analysis did not show any difference between head and neck and other parts of the body regarding prognostic value of SLN status in MCC patients. It seems that further studies with long follow-up are needed to further elucidate this issue.

### 4.2. Does SLN Mapping Actually Improves Survival in cN0 MCC Patients?

Bulk of medical literature is devoted to evaluate the accuracy of SLN mapping in MCC. However, we aimed to know if actually SLN mapping in cN0 MCC patients is associated with improved survival or not. Only four studies had enough survival analyses to answer the abovementioned clinical question and were included in the current systematic review. Three of the included studies compared operative nodal staging by SLN mapping with nodal observation only [14, 17, 18]. As shown in Table 1, all of these studies showed survival benefit of the SLN mapping compared to observation strategy (HR > 1 for DFS). Our systematic review also supported the previous findings with pooled HR of 1.64 for DFS. The reason of this finding is most likely the early diagnosis of regional nodal involvement by SLN mapping which leads to start of the adjuvant treatments in earlier stages of the disease course providing a better DFS.

On the other hand the pooled HR for OS was not statistically significant (HR = 1.08, *P* = 0.093). This is most likely due to the different design of Tarantola et al. study [3] as they reported the survival benefit of SLN mapping as compared to other nodal treatments (including regional nodal dissection, nodal radiation therapy, and observation) in cN0 MCC patients. Other nodal treatment strategies besides nodal observation can introduce a bias into the study as they can have survival benefit compared to nodal observation alone.

To sum up, it seems that operative nodal staging with SLN mapping provides survival benefit versus nodal observation in cN0 MCC patients. The survival benefit is mostly obvious for DFS. In order to evaluate the effect of SLN mapping on OS, larger studies with long follow-up are still needed.

### 4.3. Limitations

MCC is a rare tumor and many authors reported their experience in SLN mapping in this tumor with small sample size. In order to overcome this problem, we gathered the prognostic information in the literature performing survival analysis in the final sample of cases (172 patients). Although studies based on SEER database had very large sample size, the other included studies had relatively small sample size and this may limit the statistical power of our meta-analysis.

Comparison of SLN mapping with nodal observation in all but one of the included studies is another limitation of our study. Survival benefit of SLN mapping ought to be compared with other nodal treatment protocols such as prophylactic regional node dissection or irradiation. Thus far only Tarantola et al. study reported survival benefit of SLN mapping as compared to other nodal treatment methods and further multicenter large studies are needed in this regard.

The quality of the included studies can also be considered as a limitation of our study. As shown in Table 1, only two of the included studies reported the details of SLN mapping technique.

Publication bias is a major concern in all systematic reviews. We evaluated this bias by funnel plots and Egger's regression method. Although neither funnel plots nor Egger's test showed possible publication bias, the power of Egger's test is relatively low and we cannot rule out possible important publication bias in our systematic review.

## 5. Conclusion

SLN mapping can provide strong prognostic information regarding OS and DFS in cN0 MCC patients. SLN+ patients may suffer MCC-related death and recurrence more frequently per unit time than SLN− patients. Further multicenter studies with long follow-up are needed to evaluate the effect of the location of MCC.

More importantly, SLN mapping can improve DFS and possibly OS in cN0 MCC patients as compared to nodal observation. As MCC is a rare tumor, large multicenter prospective studies are still needed to validate the survival benefit of SLN mapping. Further studies ought to compare SLN mapping with other nodal treatment strategies such as prophylactic lymph node dissection and/or irradiation.

## Figures and Tables

**Table 1:** Characteristics of the included studies.

First author	Publication year	SLN mapping method	Total number of patients/mean age (year)/mean tumor size (cm)/male gender (%)/head and neck location (%)/number of patients underwent regional lymph node dissection in SLN+/SLN− patients (% of total patients)	Inclusion of patients in the study at a common point of the disease course	Duration of follow- up	Method of outcome evaluation (death or recurrence)/Blind outcome evaluation to SLN results	Adjustment for important confounding variables	Variable used for prognostication/major findings
Tarantola [3]	2013	n/a	114 (34 with SLN mapping, 80 with other regional treatments)/70.1/1.37/70/46.3/n-a/n-a	Yes, all were included at clinical stages x and II of disease	3.3 years (mean)	Death of all causes/n-a	Yes	SLN mapping versus other nodal treatments/OS in the patients underwent SLN mapping was higher, HR: 1.04 [0.51–2.15], *P* = 0.91

Kachare [14]	2014	n/a	1193 (474 with SLN mapping, 719 with nodal observation)/75.9/n-a/58.8/n-a/104 (21.9%)/n-a	Yes, all were included at clinical stages I and II of disease	Median of 21 months (0–83 months)	Death of disease/n-a	Yes	SLN mapping versus nodal observation, in addition prognostic significance of SLN pathological status was evaluated/DFS was higher in patients underwent SLN mapping, HR: 1.43 [1.01–2.05], *P* = 0.04 DFS was also higher in SLN+ as compared to SLN− patients. This part of study was duplicate of Fritsch et al. [16] study and excluded form final analysis

Bajetta [17]	2009	n/a	63 (21 with SLN mapping and 42 with nodal observation)/n-a/n-a/45/18/8 (38%)/0 (0%)	Yes, all were included at clinical stages I and II of disease	Median of 65 months	Death of disease/n-a	Yes	SLN mapping versus nodal observation/operative nodal staging with SLN biopsy, HR 3.44 [1.17–10]; *P* = 0.023) predicted better DFS

Sattler [18]	2013	n/a	47 (19 with SLN mapping and 27 with nodal observation)/70.32/n-a/26.3/31.6/n-a/n-a	Yes, all were included at clinical stages I and II of disease	Median of 20 months (2–234 months)	Death of disease, and death of all causes/n-a	Yes	SLN mapping versus nodal observation/SLN m aping predicted better DFS (HR 1.38 [0.45–4.20]) and OS 1.39 [0.25–7.76]) as compared to nodal observation. HR was calculated from the survival curves according to Parmar method

Fields [15]	2011	Combined radiotracer and blue dye	153 (45 SLN+, and 108 SLN−)/69/n-a/59/21.5/45 (29.4%)/0 (0%)	Yes, all were included at clinical stages I and II of disease	Median of 41 months	Death of disease/n-a	Yes	SLN pathologic status/SLN− status predicted better OS (HR: 1.86 [0.22–15.49], *P* = 0.56), and DFS (HR: 1.69 [0.37–7.65], *P* = 0.49) HR was calculated from the survival curves according to Parmar method

Kouzmina [19]	2013	Radiotracer in all, blue dye in 16	28 (9 SLN+ and 19 SLN−)/n-a/n-a/39.3/39.3/8 (28.5%)/0 (0%)	Yes, all were included at clinical stages I and II of disease	Mean of 3.6 years	Death of all causes/n-a	No	SLN pathologic status/SLN− status predicted better OS (HR: 4.82 [0.79–29.34], *P* = 0.08) HR was calculated from the survival curve according to Parmar method

Fritsch [16]	2014	n/a	721 (186 SLN+ and 535 SLN−)/n-a/n-a/61.7/24/n-a/n-a 12 patients were excluded from survival analysis due to less than 1 months follow-up	Yes, all were included at clinical stages I and II of disease	Median of 34 months	Death of disease/n-a	Yes	SLN pathologic status/SLN− status predicted better DFS in head and neck (HR: 2.22 [0.84–5.86], *P* = 0.1), and other parts of the body (HR: 3.01 [1.77–5.11], *P* = 0.00005) The second *P* value was calculated by CMA software

Cases in the literature with enough prognostic data	During the period of 1996–2013	Radiotracer and/or blue dye	172 (65 SLN+, and 107 SLN−)/68.6/1.84/45.3/40.1/30 (17.4%)/5 (2.9%)	Yes, all were included at clinical stages I and II of disease	Mean of 27.5 months (1–120 months)	Death of disease or death of all causes/n-a	Yes (refer to Table 2)	SLN pathologic status/SLN− status predicted better DFS in head and neck (HR: 4.09 [1.41–11.88], *P* = 0.009), and other parts of the body (HR: 1.65 [0.7–3.9], *P* = 0.15) SLN− status predicted better OS in head and neck (HR: 14.06 [1.63–121.28], *P* = 0.01), and other parts of the body (HR: 3.79 [0.96–14.6], *P* = 0.055). All analyses were performed by SPSS version 11.5

**Table 2:** Detailed survival analysis of the cases included in the individual patient analysis (*n* = 172).

Factor	Number of patients	HR for OS [95% CI]	*P* value	HR for DFS [95% CI]	*P* value
Age	Mean 68.58	1.039 [0.98–1.09]	0.13	1.003 [0.97–1.03]	0.83
Tumor size	Mean 1.85	0.59 [0.23–1.5]	0.27	1.017 [0.65–1.53]	0.93
Gender			0.212		0.55
Male	78	1.86 [0.69–5.02]		1.23 [0.62–2.44]	
Female	83	Referent		Referent	
Regional nodal treatment					
None	100	0.17 [0.04–0.72]	0.16	0.28 [0.1–0.78]	0.015
Nodal dissection	23	0.78 [0.18–3.31]	0.74	0.68 [0.22–2.14]	0.51
Radiotherapy	37	0.66 [0.11–3.99]	0.65	0.37 [0.1–1.29]	0.12
Both	12	Referent	Referent	Referent	Referent
Tumor location					
Limbs	95	0.95 [0.33–2.71]	0.93	0.73 [0.37–1.46]	0.38
Trunk	8	2.08 [0.24–17.52]	0.46	1.95 [0.56–6.77]	0.29
Head and neck	69	Referent	Referent	Referent	Referent
SLN status			0.002		0.015
Positive	65	6.13 [1.97–19.07]		2.25 [1.16–4.33]	
Negative	107	Referent		Referent	
